# Synthesis, magnetic and optical properties of core/shell Co_1-*x*_Zn*_x_*Fe_2_O_4_/SiO_2 _nanoparticles

**DOI:** 10.1186/1556-276X-6-460

**Published:** 2011-07-20

**Authors:** Emad Girgis, Mohamed MS Wahsh, Atef GM Othman, Lokeshwar Bandhu, KV Rao

**Affiliations:** 1 Solid State Physics Department, National Research Centre, 12311 Dokki, Giza, Egypt; 2 Refractories, Ceramics and Building Materials Department, National Research Centre, 12311 Dokki, Giza, Egypt; 3 Department of Materials Science, Royal Institute of Technology, Stockholm, 100 44 Sweden; 4 Advanced Materials and Nanotechnology Lab, CEAS, National Research Centre (NRC), El-Behouth Street, 12311 Dokki, Giza, Egypt

**Keywords:** nanostructures, oxides, cobalt ferrite, cobalt zinc ferrite, zinc ferrite, magnetic properties, diffuse reflectance.

## Abstract

The optical properties of multi-functionalized cobalt ferrite (CoFe_2_O_4_), cobalt zinc ferrite (Co_0.5_Zn_0.5_Fe_2_O_4_), and zinc ferrite (ZnFe_2_O_4_) nanoparticles have been enhanced by coating them with silica shell using a modified Stöber method. The ferrites nanoparticles were prepared by a modified citrate gel technique. These core/shell ferrites nanoparticles have been fired at temperatures: 400°C, 600°C and 800°C, respectively, for 2 h. The composition, phase, and morphology of the prepared core/shell ferrites nanoparticles were determined by X-ray diffraction and transmission electron microscopy, respectively. The diffuse reflectance and magnetic properties of the core/shell ferrites nanoparticles at room temperature were investigated using UV/VIS double-beam spectrophotometer and vibrating sample magnetometer, respectively. It was found that, by increasing the firing temperature from 400°C to 800°C, the average crystallite size of the core/shell ferrites nanoparticles increases. The cobalt ferrite nanoparticles fired at temperature 800°C; show the highest saturation magnetization while the zinc ferrite nanoparticles coated with silica shell shows the highest diffuse reflectance. On the other hand, core/shell zinc ferrite/silica nanoparticles fired at 400°C show a ferromagnetic behavior and high diffuse reflectance when compared with all the uncoated or coated ferrites nanoparticles. These characteristics of core/shell zinc ferrite/silica nanostructures make them promising candidates for magneto-optical nanodevice applications.

## Introduction

Synthesis of magnetic nanoparticles have been intensively pursued due to their unique functional properties and their wide variety of potential applications in high density magnetic recording [1-4], ferrofluids technology [5], biomedical drug delivery [6,7], and magnetic resonance imaging [8,9], data storage, biosensors [10], biocompatible magnetic nanoparticles for cancer treatment [11-14], and magneto-optical devices [15-17] among others.

In recent years, Spinel ferrite nanoparticles have been widely studied because of their excellent and convenient magnetic and electrical properties [18,19]. Among spinel ferrites, CoFe_2_O_4 _is of interest due to its high intrinsic coercivity (5,400 Oe) and moderate saturation magnetization (about 80 emu/g) as well as remarkable chemical stability and mechanical hardness, which makes it a good candidate for recording media [20,21]. Also, studies indicate that the magnetic properties of CoFe_2_O_4 _depend strongly on its morphology and are greatly affected by the size of the particles [22,23]. In addition, the magnetic properties of spinel structure CoFe_2_O_4 _can be altered by cation substitution. According to recent research, Zn^2+ ^substituting for Co^2+ ^in CoFe_2_O_4 _nanoparticles (Co_1-*x*_Zn*_x_*Fe_2_O_4_) exhibited improvement in properties such as excellent chemical stability, high corrosion resistivity, magneto-crystalline anisotropy, magneto-striction, and magneto-optical properties. Cobalt zinc ferrites nanoparticles have been prepared by different methods, such as co-precipitation, usual ceramic technique, microwave-hydrothermal method, and the solvothermal method [24-30].

In the present decade, core/shell structured nanoparticles have received much attention, due to their enhanced combination of optical, electronic, and magnetic properties compared to those of single-component nanomaterials [31]. Thus, coating magnetic nanoparticles with silica is becoming a promising and important approach in the development of magnetic nanoparticles for both fundamental studies as well as technological applications. Silica formed on the surface of magnetic nanoparticles could screen the magnetic dipolar attraction between magnetic nanoparticles, which improves the dispersion of magnetic nanoparticles in liquid media and protects them from leaching in an acidic environment. In addition, the core/shell structure enhances the thermal and chemical stability of the magnetic nanoparticles due to the silica shell which provides a chemically inert surface for magnetic nanoparticles in biological systems. Therefore, silica-coated magnetic nanoparticles can be easily allowed to conjugate its surface with various functional groups [32,33]. Also, the silica shell can enhance the optical properties of the nanoparticles [34]. The optical properties of the nanostructures have been investigated earlier using many techniques, among them is the diffuse reflectance spectroscopy [35].

The main objective of this study is to investigate the effect of Zn^2+ ^partially substituting for Co^2+ ^in CoFe_2_O_4 _nanoparticles (Co_1-*x*_Zn*_x_*Fe_2_O_4_; *x *= 0, 0.5, and 1) and shelling with silica on the magnetic and optical properties of the ferrite nanoparticles for a variety of magneto-optical nanodevice applications. From a synthesis point of view exploring the effect of firing temperatures (400°C, 600°C and 800°C) is of interest to investigate.

## Experimental work

The chemicals used for preparation of the samples were ferric nitrate (Fe(NO_3_)_3_·9H_2_O, Mw = 404.00 g/mol, Alpha Chemika™, Mumbai, India), cobalt (II) nitrate (Co(NO_3_)_2_·6H_2_O, Mw = 291.04 g/mol, WinLab, UK), and zinc nitrate (Zn(NO_3_)_2_·6H_2_O, Mw = 297.47 g/mol, WinLab, Laboratory chemicals reagent fine chemicals), citric acid monohydrate gritty, puriss, (C_6_H_8_O_7_·H_2_O, Mw = 210.14 g/mol, Riedel-Dehaën, Sigma-Aldrich, Labor Chemika Lien, GmbH, St. Louis, MO, USA), ammonia solution (30%), and tetraethyl orthosilicate (TEOS, C_8_H_20_O_4_Si, Mw = 208.33 g/mol, Merck Schuchardt OHG, Hohenbrunn, Germany).

CoFe_2_O_4_, ZnFe_2_O_4_, and Co_0.5_Zn_0.5_Fe_2_O_4 _nanoparticles have been prepared using modified citrate gel method [36,37]. Co(NO_3_)_2_·6H_2_O solution (0.25 M), Zn(NO_3_)_2_·6H_2_O solution (0.25 M), and Fe(NO_3_)_3_·9H_2_O solution (0.25 M) were prepared by dissolving the metal nitrates in distilled water. The prepared solutions were mixed in molar ratio of Me^2+^/Fe^3+ ^= 0.5 (Me^2+ ^= Co^2+^, Zn^2+^, and 0.5 Co^2+ ^+ 0.5 Zn^2+ ^for CoFe_2_O_4_, ZnFe_2_O_4_, and Co_0.5_Zn_0.5_Fe_2_O_4_, respectively) under constant stirring to get homogeneous solution with the heating rate of 5°C/min up to 80°C for 1 h. This mixture solution was added to the citric acid solution (0.25 M) maintaining the molar ratio between metal nitrates solution and citric acid solution as 1:1 and stirred for 2 h. Ammonia was added to reach pH equal to 7.5. Increasing the temperature during the stirring process leads to form a viscous gel. The gel was dried and fired at temperatures of 400°C, 600°C, and 800°C for 2 h to form CoFe_2_O_4 _(CF), ZnFe_2_O_4 _(ZF), and Co_0.5_Zn_0.5_Fe_2_O_4 _(CZF) nanoparticles.

Silica-coated magnetic nanoparticles were prepared using the modified Stöber method. The nanoparticles (fired at 400°C) were first treated by citric acid solution (0.01 M) under constant stirring for 1 h. The presence of citrate increases the organosilane affinity of the particle surface. These particles were separated and washed with distilled water several times. After that, the particles were redispersed in a mixture of absolute ethanol (80 ml) and distilled water (20 ml) the ammonia was added to the solution as a catalyst. Subsequently, 6 ml of TEOS was injected to the above solution, drop by drop at constant stirring for 24 h at room temperature to ensure the hydrolysis, after that, the condensation of TEOS on the surface of nanoparticles was achieved. Finally, the core/shell CoFe_2_O_4_/SiO_2_, Co_0.5_Zn_0.5_Fe_2_O_4_/SiO_2_, and ZnFe_2_O_4_/SiO_2 _particles were separated using external magnet, and washed with ethanol and water several times. The samples have been dried at 40°C for 24 h and fired at temperatures 400°C, 600°C, and 800°C, respectively, for 2 h.

The morphology of uncoated and coated nanoparticles was studied using transmission electron microscopy, TEM (JEOL 1230, JEOL, Tokyo, Japan). The phase composition and average crystallite size of the core/shell ferrite nanoparticles were investigated using X-ray diffractometer (Model Bruker D8 Advance (Bruker AXS, Madison, WI, USA), Cu-Kα1 (*λ *= 1.54058 Å) radiation with secondary monochromator at a scanning speed of 1°/min). In addition, vibrating samples magnetometer (model is Princeton FM-1, Princeton Applied Research, Oak Ridge, TN, USA) and UV/VIS double-beam spectrophotometer (model is no. Lambda 35, Perkin Elmer, Waltham, MA, USA) were used to measure the magnetic properties and diffuse reflectance of the prepared ferrite nanoparticles, respectively.

## Results and discussion

**Figure 1:**
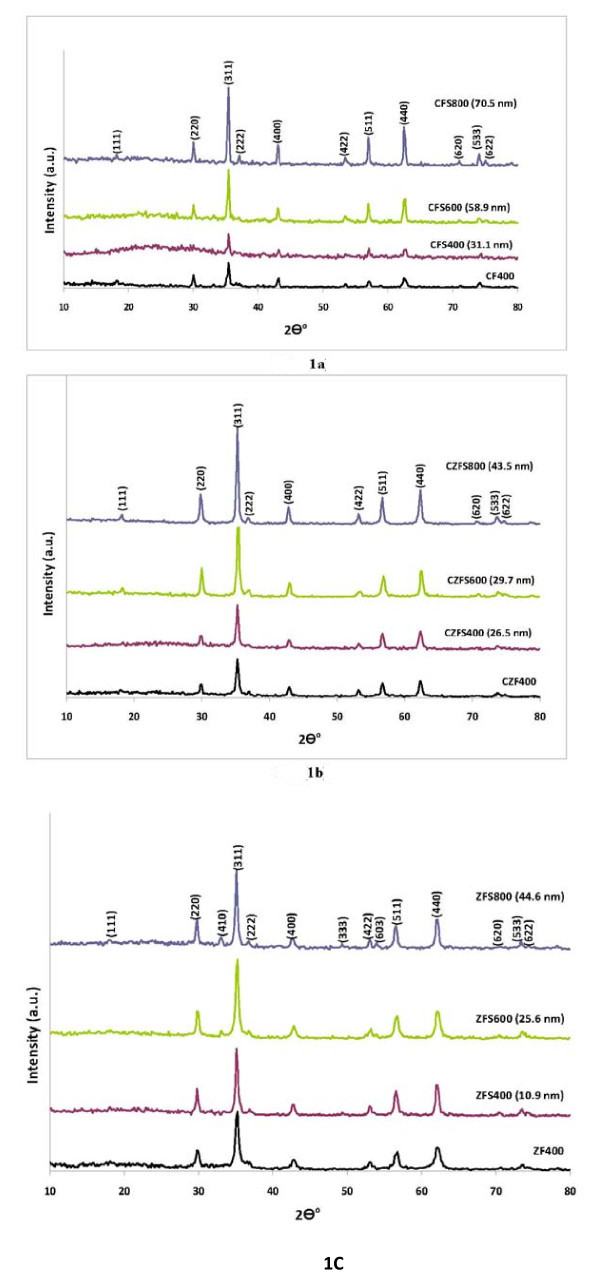
**XRD patterns of core/shell CoFe_2_O_4_/SiO_2 _(a), Co_0.5_Zn_0.5_Fe_2_O_4_/SiO_2 _(b), and ZnFe_2_O_4_/SiO_2 _(c) nanoparticles**.

Figure 1a, b, c shows the X-ray diffraction patterns of core/shell Co_1-*x*_Zn*_x_*Fe_2_O_4_/SiO_2 _nanoparticles, in which *x *= 0, 0.5, and 1, respectively. All the strong peaks appeared at 2*θ *= 18.4°, 30.084°, 35.437°, 37.057°, 43.058°, 53.445°, 56.973°, 62.585°, 70.78°, 74.009°, and 75.00° are indexed to the crystal plane of spinel ferrite (Co_1-*x*_Zn*_x_*Fe_2_O_4_) structure (111), (220), (311), (222), (400), (422), (511), (440), (620), (533), and (622), respectively. In addition, the intensities of the peaks are found to increase by increasing the firing temperature due to the increase of the crystalline phase. From Figure 1a, b, it was observed that the X-ray diffraction patterns (XRD) of Co_0.5_Zn_0.5_Fe_2_O_4 _nanoparticles having the same crystal plane of CoFe_2_O_4 _nanoparticles which confirms the formation of the good spinel structure. In addition, no secondary phase was detected in XRD patterns which ensure the purity of the Co_0.5_Zn_0.5_Fe_2_O_4 _nanoparticles. The average crystallite size of Co_1-*x*_Zn*_x_*Fe_2_O_4_/SiO_2 _nanoparticles were estimated using the Scherrer's formula; *D *= 0.9*λ*/(FWHM × cos *θ*), where *D *is the crystallite size; FWHM is the observed full width at half maximum; *θ *is the Bragg angle, and *λ *is the wavelength of the X- ray radiation (*λ *= 1.54058 Å). In addition, a broad peak at 2*θ *approximately 22-25° has been detected in the samples coated with silica shell and fired at 400°C for 2 h as shown in Figure 1a. This broad peak is due to the presence of the amorphous silica. By increasing the firing temperature, amorphous silica starts to disappear and only the diffraction peaks of spinel ferrite Co_1-*x*_Zn*_x_*Fe_2_O_4 _phase were detected due to the formation of good core/shell structure. Figure 1c shows the XRD pattern of the zinc ferrite/silica nanoparticles (ZFS) fired at 800°C. Similar phases have been observed as mentioned above except for the presence of three weak diffraction peaks at 2*θ *= 33.194°, 48.94°, and 54.06° corresponding to (410), (333), and (603) crystal planes of rhombohedral zinc silicate phase. The latter phase arises because of solid state reaction of ZnO resulting from small dissociation of ZnFe_2_O_4 _core at high temperature (800°C) with SiO_2 _shell forming Zn_2_SiO_4 _phase.

**Figure 2:**
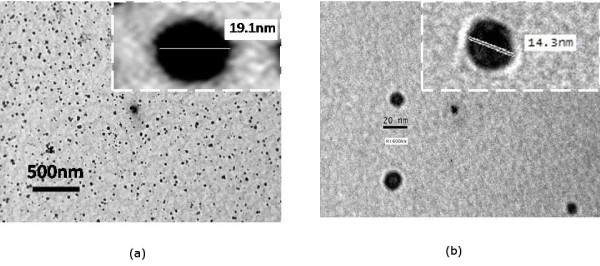
**TEM micrographs of ZnFe_2_O_4 _nanoparticles uncoated (a) and coated with silica (b) fired at 400°C**.

Figure 2 shows the TEM images of zinc ferrite nanoparticles uncoated and coated with silica shell fired at 400°C. It was observed that the estimated average particle size of the zinc ferrite and zinc ferrite/silica nanoparticles varies between 12 and 14 nm.

**Figure 3:**
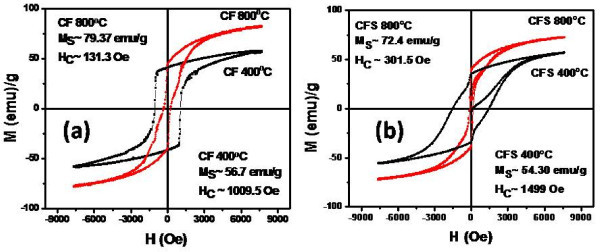
**Hysteresis loops of CoFe_2_O_4 _nanoparticles uncoated (a) and coated with silica shell (b)**.

The hysteresis loops and the magnetic parameters (saturation magnetization (Ms) and switching field (Hc)) of the prepared ferrite nanoparticles fired at 400°C and 800°C were measured at room temperature (27°C) using vibrating samples magnetometer. Figure 3a shows the hysteresis loops of uncoated cobalt ferrite nanoparticles fired at 400°C and 800°C. It is clear that by increasing the firing temperature from 400°C to 800°C, the Ms increased from 56.7 to 79.37 emu/g and the Hc decreased from 1009.5 to 131.3 Oe. Increasing the firing temperature leads to increase the crystal size of the ferrite nanoparticles which reflects on the magnetization state by creating a multidomains state instead of single-domain state. Multidomains need less magnetic field to switch compared with the single domain state. Accordingly, it was found that at large crystallite size, the switching field decreases and the magnetization saturation increases compared with the smaller size. Figure 3b shows the hysteresis loops of the coated cobalt ferrite nanoparticles fired at 400°C and 800°C where a slight decrease in the saturation magnetization compared with the uncoated nanoparticles was observed. The slight decrease in the magnetization saturation and increase in the switching field is due to the coating effect, where each particle was separated from its neighbors with silica shell which leads to decrease the magnetostatic coupling between the particles. By increasing the firing temperature to 800°C, the crystals will grow leading to increase the magnetization saturation and create a multidomains state.

On the other hand, the hysteresis loop is much wider for the cobalt ferrite samples coated with silica shell (CFS) and fired at 400°C compared with cobalt ferrite samples fired at 800°C. This confirms that by increasing the firing temperature, the crystallite size increases leading to decrease of the switching field. Also, it was found that, for the cobalt ferrite nanoparticles coated with silica (CFS), the magnetic moment increases with increasing the firing temperature from 400°C to 800°C. As mentioned earlier from the XRD analysis, with increasing the firing temperature, the amorphous silica starts to disappear and the diffraction peaks of spinel cobalt ferrite phase only are found at higher temperatures due to the formation of robust core/shell structure (Figure 1a). This leads to creation of a very thin layer of cobalt ferrite silicate at the surface of these cobalt ferrite nanoparticles which decrease the effect of the amorphous silica shell and hence increase the magnetic moment at higher firing temperature.

**Figure 4:**
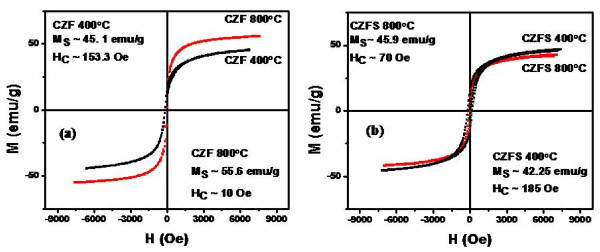
**Hysteresis loops of Co_0.5_Zn_0.5_Fe_2_O_4 _nanoparticles uncoated (a) and coated with silica shell (b)**.

The hysteresis loops of cobalt zinc ferrite nanoparticles (Co_0.5_Zn_0.5_Fe_2_O_4_) uncoated and coated fired at 400°C and 800°C are shown in Figure 4. When the Co^2+ ^ions in cobalt ferrite samples is substituted by Zn^2+ ^ions (Co_0.5_Zn_0.5_Fe_2_O_4 _and ZnFe_2_O_4_), the magnetization saturation and the switching field are found to decrease with increasing the concentration of Zn^2+ ^ions. Accordingly, the width of hysteresis loop and the magnetic moment decrease due to the substitution of the magnetic Co element by Zn element which is a non-magnetic material. Core/shell ferrite nanoparticles show lower magnetization saturation than the uncoated ferrite nanoparticles fired at the same temperature, while the switching field increases for the coated ferrite nanoparticles. This is due to the effect of silica shell coating where each particle was separated from its neighbors by the shell layer leading to decrease the magnetostatic coupling between the particles.

**Figure 5:**
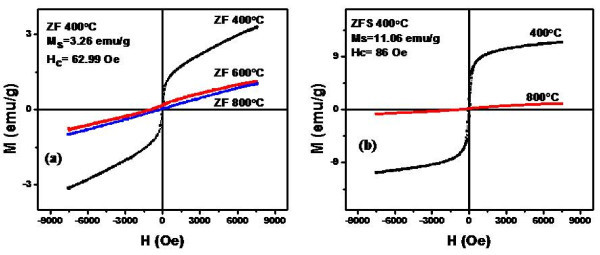
**Hysteresis loops of ZnFe_2_O_4 _nanoparticles uncoated (a) and coated with silica shell (b)**.

Figure 5a shows the hysteresis loops of uncoated zinc ferrite samples fired at 400°C, 600°C, and 800°C. It is clear that the zinc ferrite nanoparticles fired at 400°C show a ferromagnetic behavior while by increasing the firing temperature to 600°C, the magnetization state of the zinc ferrite nanoparticles starts to transfer from the ferromagnetic state to the paramagnetic state. With the increase of the firing temperature up to 800°C the hysteresis loop of the zinc ferrite nanoparticles shows a typical paramagnetic behavior.

Figure 5b shows the hysteresis loops of core/shell zinc ferrite nanoparticles coated with silica shell (ZFS) fired at 400°C and 800°C. It is clear that at 400°C, the zinc ferrite/silica nanoparticles show a ferromagnetic behavior compared with the sample fired at 800°C which shows a paramagnetic behavior.

From X-ray diffraction results (Figure 1c), it is clear that there are three weak diffraction peaks corresponding to crystal planes of rhombohedral zinc silicate (Zn_2_SiO_4_) phase were observed. The latter phase appears due to solid state reaction of ZnO resulting from small dissociation of ZnFe_2_O_4 _core at high temperature (800°C) with SiO_2 _shell leading to form Zn_2_SiO_4 _phase. The Zn_2_SiO_4 _phase has no magnetic property. This explains the transformation of the magnetization state from ferromagnetic state to paramagnetic state with the increase of the firing temperature from 400°C to 800°C. The Ms and Hc values of the prepared coated and uncoated ferrite nanoparticles are summarized in Table 1.

**Table 1:** Summary of the magnetization saturation and switching field (H_C_) values at room temperature (27°C)

Sample code	M_S _(emu/g)	H_C _(Oe)
CF 400	56.7	1,009.5
CF 800	79.37	131.3
CFS 400	54.3	1,499
CFS 800	72.4	301.5
CZF 400	45.1	153.3
CZF 800	55.6	10
CZFS 400	42.25	185
CZFS 800	45.9	70
ZF400	3.26	62.99
ZFS 400	11.06	86

**Figure 6:**
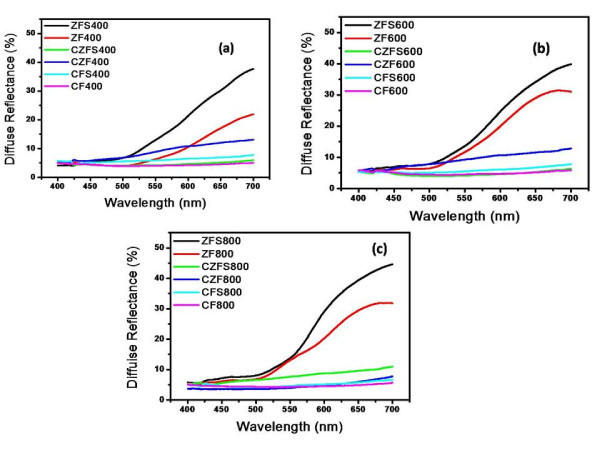
**Diffuse reflectance spectra of core/shell nanoparticles fired at 400°C (a), 600°C (b), and 800°C (c)**.

Figure 6 shows the diffuse reflectance spectra of various cobalt ferrite, zinc ferrite, and cobalt zinc ferrite nanoparticles uncoated and coated with silica shell which were fired at 400°C (Figure 6a), 600°C (Figure 6b) and 800°C (Figure 6c). It is clear that zinc ferrite nanoparticles coated with silica shell exhibit the highest value of diffuse reflectance percentage compared with all core/shell ferrite samples. In addition, the diffuse reflectance percentage of zinc ferrite nanoparticles coated with silica increases by increasing the firing temperature from 400°C (37.4%) up to 800°C (44.64%). The diffuse reflectance percentage of uncoated zinc ferrite nanoparticles, fired at 400°C, 600°C and 800°C decreased compared with zinc ferrite nanoparticles coated with silica shell. This is attributed to the effect of silica shell, which enhances the optical properties of core/shell ferrite nanoparticles. On the other hand, cobalt ferrite nanoparticles show a very low diffuse reflectance compared with the other prepared nanoparticles (zinc ferrite and cobalt zinc ferrite nanoparticles). This is due to the effect of the change of color on the optical properties of the ferrite nanoparticles from black at CoFe_2_O_4_, to brown at Co_0.5_Zn_0.5_Fe_2_O_4 _and to orange at ZnFe_2_O_4 _by increasing the Zn^2+ ^ions which substitute the Co^2+ ^ions (Co_1-*x*_Zn*_x_*Fe_2_O_4_). In addition, the presence of the silica shell plays an important role in the optical properties enhancement of the prepared core/shell ferrite samples. When a beam of incident light impinges on the surface of these core/shell nanoparticles, only a small fraction is specularly reflected, while the remainder penetrates into the mass and undergoes scattering (multiple reflections, refractions, and diffraction in all directions) as well as wavelength-dependent absorption within the colored material (diffused rays will lose some wavelengths during their walk in the material, and will emerge colored). Part of this radiation ultimately leaves the mass in all directions and constitutes so-called diffusely reflected light [38].

**Figure 7:**
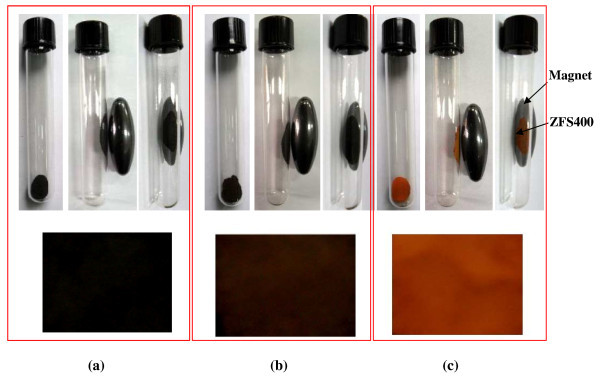
**Photographs of CoFe_2_O_4_/SiO_2 _(a), Co_0.5_Zn_0.5_Fe_2_O_4_/SiO_2 _(b), and ZnFe_2_O_4_/SiO_2 _(c) nanoparticles fired at 400°C**.

Figure 7 shows the photographs of CoFe_2_O_4_/SiO_2 _(Figure 7a), Co_0.5_Zn_0.5_Fe_2_O_4_/SiO_2 _(Figure 7b), and ZnFe_2_O_4_/SiO_2 _(Figure 7c) core/shell ferrite nanoparticles fired at 400°C for 2 h, with and without an external magnet effect. It can be seen that all the core/shell nanoparticles show manifestations of ferromagnetic behavior as shown in the photographs where the nanoparticles were attracted to the external magnet. Also, it is clear that the nanoparticles colors were changed from black (CoFe_2_O_4_/SiO_2_), to brown (Co_0.5_Zn_0.5_Fe_2_O_4_/SiO_2_), and to orange (ZnFe_2_O_4_/SiO_2_) by increasing the Zn^2+ ^ion substituting for Co^2+ ^ions.

## Conclusion

Core/shell Co_1-*x*_Zn*_x_*Fe_2_O_4_/SiO_2 _(*x *= 0, 0.5, and 1) nanoparticles were prepared using modified citrate gel technique and coated with silica shell. The samples have been fired at 400°C, 600°C, and 800°C, respectively. It is concluded that cobalt ferrite nanoparticles fired at 800°C showed the highest magnetic properties, while zinc ferrite nanoparticles coated with silica and fired at 800°C shows the best enhanced optical properties. X-ray diffraction patterns show the presence of spinel ferrite crystalline phase as the main phase in all prepared core/shell ferrite nanoparticles. In addition, the average crystallite size increases on increasing the firing temperature from 400°C up to 800°C. Zinc ferrite nanoparticles coated with silica shell and fired at 400°C show a ferromagnetic behavior and high diffuse reflectance compared with all uncoated and coated nanoparticles due to the presence of zinc ions and the silica shell which play an important role on the optical properties enhancement. The firing temperatures as well as the crystallite size parameters have great effect on the magnetic and the optical properties of core/shell ferrite nanoparticles. Core/shell ferrite nanoparticles coated with silica are found to enhance the optical properties of the magnetic nanoparticles. Core/shell zinc ferrite nanoparticles coated with silica shell and fired at 400°C show promising results for photo-magnetic nanodevice applications and for magneto-optical recording industry.

## Competing interests

The authors declare that they have no competing interests.

## Authors' contributions

EG participated in the design of the study, measured and explained the magnetic properties & SEM images and contributed in the writing of the manuscript. MMSW participated in the conception and design of the study, prepared the nanoparticles, explained the XRD analysis & diffuse reflectance spectra and contributed in the writing of the manuscript. AGMO participated in the explanation of the XRD analysis and helped to draft the manuscript. LB participated in the measurement of magnetic properties. KVR participated in the design and coordination and helped to draft the manuscript. All authors read and approved the final manuscript.
